# A probabilistic method to estimate the burden of maternal morbidity in resource-poor settings: preliminary development and evaluation

**DOI:** 10.1186/1742-7622-11-3

**Published:** 2014-03-13

**Authors:** Edward Fottrell, Ulf Högberg, Carine Ronsmans, David Osrin, Kishwar Azad, Nirmala Nair, Nicolas Meda, Rasmane Ganaba, Sourou Goufodji, Peter Byass, Veronique Filippi

**Affiliations:** 1 UCL Institute for Global Health, University College London, 30 Guilford Street, London WC1N 1EH, United Kingdom; 2 Umeå Centre for Global Health Research, Department of Public Health and Clinical Medicine, Umeå University, Umeå, Sweden; 3 Department of Women’s and Children’s Health, Uppsala University, Academic Hospital, 751 85 Uppsala, Sweden; 4 London School of Hygiene and Tropical Medicine, Keppel Street, London WC1E 7HT, United Kingdom; 5 Perinatal Care Project, Diabetic Association of Bangladesh (BADAS), BIRDEM 122 Kazi Nazrul Islam Avenue Shahbagh, Dhaka 1000, Bangladesh; 6 Ekjut, Plot 556B, Potka, Chakradharpur Pin - 833102, West Singhbhum, Jharkhand, India; 7 Centre MURAZ, Ministry of Health, 01 PO Box 390, Bobo-Dioulasso 01, Burkina Faso; 8 Centre de Recherche en Reproduction Humaine et en Démographie, Cotonou, Benin

**Keywords:** Maternal health, Morbidity, Developing countries, Pregnancy, Childbirth, Bayesian analysis, Africa, Asia

## Abstract

**Background:**

Maternal morbidity is more common than maternal death, and population-based estimates of the burden of maternal morbidity could provide important indicators for monitoring trends, priority setting and evaluating the health impact of interventions. Methods based on lay reporting of obstetric events have been shown to lack specificity and there is a need for new approaches to measure the population burden of maternal morbidity. A computer-based probabilistic tool was developed to estimate the likelihood of maternal morbidity and its causes based on self-reported symptoms and pregnancy/delivery experiences. Development involved the use of training datasets of signs, symptoms and causes of morbidity from 1734 facility-based deliveries in Benin and Burkina Faso, as well as expert review. Preliminary evaluation of the method compared the burden of maternal morbidity and specific causes from the probabilistic tool with clinical classifications of 489 recently-delivered women from Benin, Bangladesh and India.

**Results:**

Using training datasets, it was possible to create a probabilistic tool that handled uncertainty of women’s self reports of pregnancy and delivery experiences in a unique way to estimate population-level burdens of maternal morbidity and specific causes that compared well with clinical classifications of the same data. When applied to test datasets, the method overestimated the burden of morbidity compared with clinical review, although possible conceptual and methodological reasons for this were identified.

**Conclusion:**

The probabilistic method shows promise and may offer opportunities for standardised measurement of maternal morbidity that allows for the uncertainty of women’s self-reported symptoms in retrospective interviews. However, important discrepancies with clinical classifications were observed and the method requires further development, refinement and evaluation in a range of settings.

## Background

The aim of most safe motherhood programmes in resource-poor settings is to reduce maternal mortality and morbidity. There is great interest from funders, policy makers and researchers in evaluating their success using health outcomes, particularly a reduction in deaths or severe complications. Measurement of maternal mortality in these settings is notoriously elusive, however, given its relative rarity, the large sample sizes needed and the reliance on verbal autopsy methods to identify pregnancy status and causes of death when it occurs at home [1]. For every maternal death there are a large number of women who suffer illness and may come close to death and suffer long-term consequences of obstetric morbidity [2-4]. Population-based estimates of the burden of maternal morbidity, therefore, could be useful indicators for monitoring trends, priority setting and evaluating the health impact of interventions [5,6], particularly within a context of falling maternal mortality. Measuring the burden of maternal morbidity is also difficult, however, particularly in populations where many women deliver at home and may go through pregnancy and the post-partum period with limited contact with health services.

Data on a sample of hospital users are unlikely to provide a representative picture of all maternal morbidity at the population level, although new methods do show promise for extreme, life-threatening conditions [7]. Furthermore, women’s ability to accurately recall and report signs or symptoms related to diagnoses of complications is limited, mostly because of lack of specificity, thereby leading to difficulties in estimating prevalence [8-11]. Thus, retrospective interviews on women’s perceived obstetric complications tend to over-estimate the burden and evaluations of various approaches have generally shown poor validity [9].

In general, community-based survey approaches have relied on reports of the presence or absence of specific signs and symptoms in order to ascertain a definitive binary outcome (whether or not the woman experienced a specific complication). Clinical review of the data, with or without decision tree algorithms, may be used to classify cases into specific morbidity cause categories [8]. However, such approaches do not generally account for uncertainty of lay recall and reporting of signs, and the derived morbidity diagnoses based on binary classifications may falsely imply certainty of classification. Forced dichotomy of the outcome based on uncertain symptom histories may partly explain the over-estimates of morbidity based on these methods.

Identifying multiple possible complications and their causes with specified degrees of likelihood, which may then be aggregated to provide a profile of cause-specific morbidity burdens at the population level, may be a more realistic endeavour than seeking crude binary classifications based on self-reports of questionable validity. This paper describes the development and preliminary evaluation of an innovative probabilistic approach to handling women’s self-reports of pregnancy and delivery experiences, signs and symptoms, to estimate population-level burdens of obstetric morbidity and its causes as needed by local health managers and researchers in resource-and data-poor settings.

## Methods

### Theory & technical overview

There are three initial steps in developing the probabilistic method, hereafter called InterSAMM (***Inter*** *preting* ***S*** *evere* ***A*** *cute* ***M*** *aternal* ***M*** *orbidity*). Step 1 requires selection of a finite list of signs and symptoms (collectively termed 'indicators’) and morbidity cause categories that should be included; step 2 involves estimation of *a priori* probabilities of occurrence of each morbidity cause and indicator among all pregnant women; and step 3 requires estimation of the probability of each indicator given the presence of specific morbidity. Step 1 resulted in 72 indicators and 10 cause categories (Table 1) selected on the basis of previous surveys of obstetric morbidity, realistic expectations of what could be measured through surveys and discussions during a one-day workshop of maternal health experts in London in September 2009. The list of indicators included is not intended to be specific to any one questionnaire, but rather to be adaptable to various questionnaires that may be in use across the world.

**Table 1:** List of the 72 signs and symptoms (collectively called 'indicators’) and the 10 direct and indirect causes of obstetric complications included in the InterSAMM probabilistic model

**Indicators**			**Cause categories**
1. aged under 20 yrs	25. any diagnosis of anaemia	49. did she visit more than one health facility	Puerperal Infection
2. aged 20 to 34 yrs	26. any pallor	50. intent to deliver at home	Antepartum Haemorrhage
3. aged 35 yrs or more	27. any jaundice or yellow eyes	51. intend to deliver at home but delivered in facility	Postpartum Haemorrhage
4. was this her first pregnancy	28. any cyanosis or blue lips	52. any acute abdominal pain before labour	Pre-eclampsia
5. has she had 2 to 4 pregnancies	29. was baby delivered alive	53. any acute abdominal pain after delivery	Eclampsia
6. were there >4 previous pregnancies	30. was baby delivered dead	54. any previous c-section	Obstructed Labour
7. was this a multiple pregnancy	31. was baby's position abnormal	55. genital infection/foul smelling discharge pp*	Uterine Rupture/Pre-rupture
8. any attempt to terminate this pregnancy	32. major bleeding in 1st 3 months of pregnancy	56. leaking membranes before labour start	Anaemia
9. was she <5 months pregnant at end of pregnancy	33. major bleeding >3m & before labour	57. any augmentation of labour	Malaria
10. any IV or IM antibiotics required	34. major bleeding during labour	58. any persistent fever>3 wks	Other infections
11. any blood transfusion required	35. major bleeding after delivery	59. any swollen glands	
12. any blood transfusion received	36. was blood pressure raised during pregnancy	60. did she require iron injections	
13. was she bedbound for more than 1 day pp*	37. was delivery by forceps/ventouse	61. any swelling of face	
14. breathless carrying out normal activities ap*	38. was delivery by Caesarean	62. any blurred vision	
15. breathless carrying out normal activities pp*	39. was delivery at home	63. any severe headache before labour	
16. any loss of consciousness	40. was delivery at a health facility	64. any severe headache after delivery	
17. any acute fever before pregnancy end	41. were fits only pregnancy related	65. any history of migraine	
18. any acute fever after pregnancy end	42. was labour prolonged >24 hrs	66. any diagnosis of haemorrhage	
19. any recurrent fever	43. was labour prolonged >48hrs	67. any diagnosis of hypertension	
20. any shivering with fever	44. was delivery of the placenta delayed	68. any diagnosis of malaria	
21. did she ever have fits	45. was there manual removal of the placenta	69. any diagnosis of infection	
22. did she have a diagnosis of epilepsy	46. had professional assistance at delivery	70. any diagnosis of rupture	
23. any hysterectomy	47. intention to deliver at health facility	71. was delivery said to be uncomplicated	
24. haemoglobin less than 8g/dl	48. abnormal proteinuria reported	72. self-reported delivery complication	

*pp = postpartum; ap = antepartum.

This work builds on previous work on verbal autopsy methods described in detail elsewhere [12]. Completion of steps 1-3 above allows the application of Bayes’ theorem whereby the probability of severe acute maternal morbidity in general, and of each specific maternal morbidity cause category in Table 1, can be determined given the presence of specific self-reported signs, symptoms or indicators: in mathematical terms P(C|I). Associated with each indicator (I) and each morbidity (C) is the probability of occurrence among all pregnant or recently delivered women approximated *a priori* in step 2 using a semi-qualitative scale [12]. The *a priori* estimate of the baseline probability of any woman reporting an indicator (P(I)) can reflect the sensitivity, specificity and reliability of women’s self-reports of specific symptoms. Baseline probabilities of reporting a specific indicator given the presence of a specific morbidity (P(I|C)), estimated *a priori* in step 2, can then moderate the association between commonly over-reported signs and symptoms and specific diagnoses. For example, reports of bleeding do not definitively lead to a diagnosis of postpartum haemorrhage, but rather increase its likelihood relative to other morbidities. Similarly, each reported indicator adjusts the overall likelihood of severe near-miss morbidity in general, and each specific cause of morbidity, raising it or lowering it until, when all indicators have been processed, the likelihoods are known for each cause for each individual case. A hypothetical illustration is shown in Table 2, in which the unconditional baseline likelihoods of morbidity and specific causes are shown in row 1 and subsequent rows reflect modified likelihoods for each reported indicator in turn until, finally, the likelihood of severe near-miss morbidity is 92% and the most likely causes are cause 3 (69%) and cause 4 (23%). By simultaneously adjusting the probability of each of the finite list of morbidity causes according to affirmative answers to specific indicators, InterSAMM calculates the likelihood of each cause for each individual. These likelihoods can then be aggregated to give population-level estimates of the likelihood of morbidity and its specific causes.

**Table 2:** Hypothetical example of probabilistic interpretation of lay-reported indicators of morbidity

**Indicator**	**Probability of selected causes of death**				
	**Near-miss morbidity**	**Cause 1**	**Cause 2**	**Cause 3**	**Cause 4**
Unconditional probability	0.15	0.05	0.10	0.30	0.40
Indicator 1	0.93	0.01	0.01	0.44	0.36
Indicator 2	0.94	0.01	0.02	0.48	0.40
Indicator 3	0.92	0.02	0.01	0.69	0.23

The example shows how each reported indicator in this single case affects the cause probability. In this case, the conclusion is an overall likelihood of morbidity of 92%, with Cause 3 being the most likely cause, with a likelihood of 69%.

### The computer program

A computer program applying the above principles was written in Microsoft Visual FoxPro software. As illustrated in Table 2, the computer program was designed to calculate the likelihood of all-cause near-miss morbidity status separate from the likelihoods of specific causes. The concept of morbidity encompasses a spectrum of conditions that can range from very mild to life-threatening. Mild, short-term morbidity is likely to be common, but may not be of clinical significance in terms of the conditions listed in Table 1 and may be of limited public health relevance in low-income settings. Therefore, a cut-off of 30% likelihood of maternal morbidity was selected a priori, below which cases are considered to be non-morbid and the probabilities of specific causes are set to zero. The application of cut-off points to a continuous likelihood distribution is somewhat arbitrary. Nevertheless, categorical classification is useful in terms of conceptualising the severity of morbidities in clinical terms and is necessary for comparison with clinical classifications which typically uses exclusive binary categorisations of outcomes. To further enable categorical classification of cases, it is reasonable to assume that individuals whose reported symptoms result in a likelihood of near-miss in excess of 90% are at the extreme end of the morbidity spectrum whilst lower likelihoods (30% to 90%) are likely to represent clinically significant but not necessarily immediately life-threatening conditions.

For morbid cases (with a probability of near-miss above 30%), the InterSAMM computer program displays the probability of specific causes and multiple causes can be assigned to each case. Certain rules are applied to limit the number of specific causes reported. First, the likelihood of all determinate causes must have increased by an appreciable and decisive amount, defined as the square root of the unconditional cause probability. If this condition is not met for any cause due to insufficient indicators being available the cause will be categorised as 'indeterminate’. For multiple causes to be reported, each cause likelihood must fall within 50% of the previous, more likely, cause.

To handle multiple causes, each individual case can be split between multiple causes proportional to the likelihood of each determinate cause. For example, if an individual is assigned two causes, haemorrhage and anaemia, with likelihoods of 50% and 40%, respectively, 0.5 will be added to the total population count of haemorrhage, 0.4 will be added to anaemia and 0.1 (the remainder of 100% of this case) will be added to the population count of cause uncertainty. When the population counts of each cause category are divided by the total number of cases, one gets the population cause-specific morbidity fraction (CSMF) attributable to each cause category and an indication of overall uncertainty of cause diagnoses.

### Development & refinement

**Figure 1:**
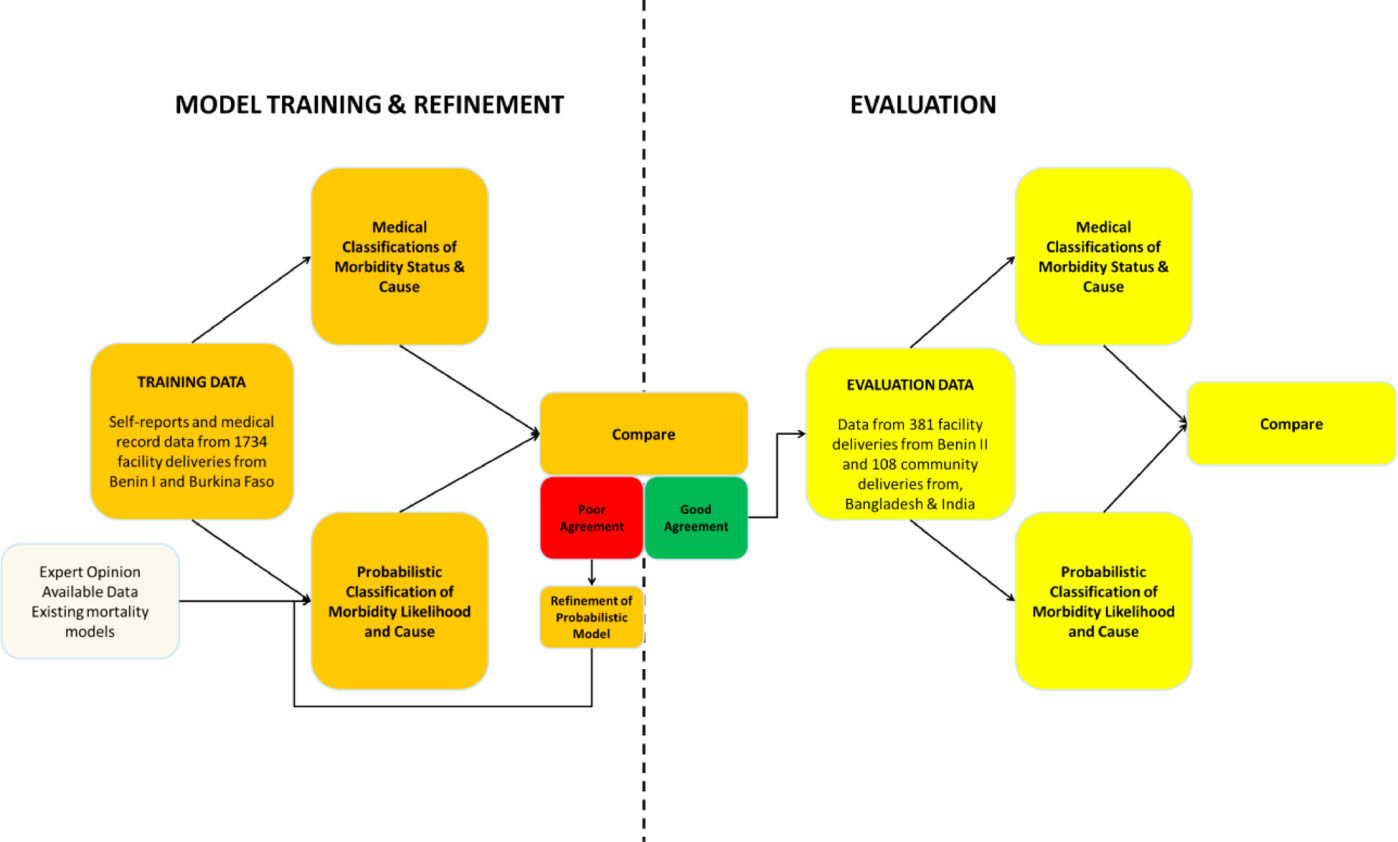
Summary of the InterSAMM development and evaluation process using data sources from Benin, Burkina Faso, Bangladesh and India.

The amber boxes in Figure 1 summarise the initial development, training and refinement of the InterSAMM probabilistic method. Data from medical records combined with self-reports of 1734 pregnancy and delivery experiences collected as part of two separate prospective cohort studies of women recruited in hospitals in Benin (*Benin I data*) and Burkina Faso were used to develop, test and refine the probabilistic model. The studies are described in detail elsewhere [5,6,13], but essentially all women with severe obstetric complications from the hospital sampling frames were selected using clinical indications (such as haemorrhagic shock) and case-management procedures (such as hysterectomy) and approximately two uncomplicated deliveries were selected for every complicated case. This sample therefore represents a higher-risk population, with a much greater burden of morbidity than would be expected in a population-based sample. The selected cases represent only the extreme ends of the morbidity scale: near-miss and uncomplicated deliveries.

Relevant morbidity signs and symptoms as recorded in case notes and reported by women themselves were extracted from the data and formatted to be used with InterSAMM. The population distribution of near-miss likelihoods, morbidity categorisations and cause distributions were compared with clinical classifications. Clinical diagnoses of multiple causes per case were split evenly between determinate causes, and fractions of each cause were then summed and divided by the total number of cases to calculate clinician-derived population-level CSMFs. This approach approximates the method used to handle multiple causes derived from InterSAMM, with two important differences. Firstly, cases must be split evenly between determinate causes because no quantification of likelihood of each cause is available and no assumptions of hierarchy can be assumed, even if it is likely that clinicians might consider certain causes to have a greater significance or contribution to morbidity than others. Secondly, any sense of uncertainty that clinicians had in assigning causes has been lost and cannot be accounted for.

Comparisons between clinical classifications and results from InterSAMM were carried out. An iterative process of comparisons with clinical classifications and refinements of *a priori* probabilities and the probabilistic model was followed, illustrated by a loop of refining the probabilistic model, re-running the data and comparing the results in Figure 1. This process enabled data-driven refinements to the *a priori* probabilities to produce a final probabilistic model that handled indicators to estimate morbidity and cause likelihood distributions comparable to clinical classifications.

### Evaluation

The yellow boxes in Figure 1 illustrate the evaluation component of the study whereby additional datasets of 381 hospital deliveries from a different study in Benin (*Benin II data*) [8], and a purposive sample of 57 deliveries from a community-based cohort from Bangladesh [14] and 51 deliveries from a community-based cohort from India [15] were used to evaluate the model. The Benin II data were collected during a hospital-based validation study of an obstetric morbidity questionnaire whereby a stratified sample of women with and without maternal morbidity were identified retrospectively from case notes using criteria to define near-miss and less severe morbidity that the investigators themselves derived and described elsewhere [8].

The data from Bangladesh and India are population based and are a sample of women’s self-reports of their pregnancy and delivery experiences collected through interviews with mothers in their homes following the end of pregnancy as part of cluster randomised controlled trials of community-mobilisation interventions to improve maternal and neonatal outcomes. The samples from Bangladesh and India were purposefully selected to represent a range of reported morbidities and case histories, each of which was reviewed by an experienced physician who assigned likely causes of morbidity to each case.

In all evaluation data the case-mix was heavily skewed towards the morbid end of the spectrum or high-risk populations. An important difference between the evaluation datasets and the training dataset from Benin I and Burkina Faso described previously is that reports of signs and symptoms used by InterSAMM come only from women’s self-reports–hospital record data were not used as an input to the probabilistic model during the evaluation phase. InterSAMM’s performance was evaluated in terms of comparability of SAMM classifications and population CSMFs with clinical classifications. All comparisons were based on mapping and reconciliation of the range of terminologies used by clinicians to describe causes into the cause categories used by the probabilistic method. Causes identified by clinicians that did not fit into any InterSAMM cause category were grouped together as “other causes”.

## Results

### Development & refinement

**Figure 2:**
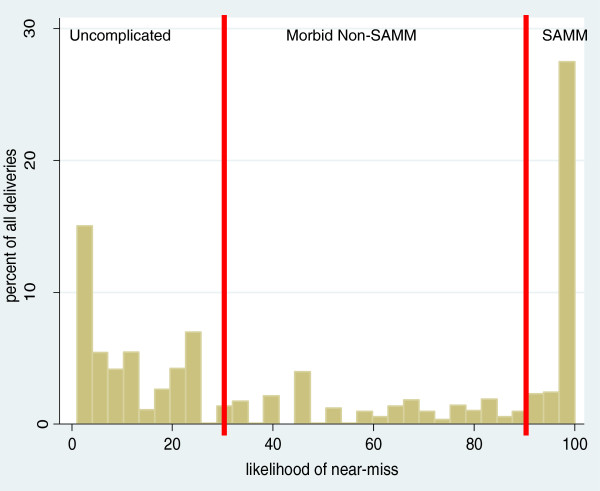
**Distributions of likelihoods of severe acute maternal morbidity (SAMM) for 1734 deliveries from Benin and Burkina Faso according to the InterSAMM probabilistic method.** Vertical red lines illustrate cut-offs between 'uncomplicated’, 'morbid non-SAMM’ and 'SAMM’ categorisations.

Data were available for 41 of the InterSAMM indicators in the combined Benin and Burkina Faso 'training’ dataset. The average number of indicators per case was 8 (minimum 3; maximum 17). Out of the 1734 deliveries from Benin and Burkina Faso, 611 (35%) were classified as SAMM by clinicians and the remaining 65% were classified as uncomplicated deliveries. Figure 2 shows the distribution of SAMM likelihoods for all 1734 deliveries. Considering cases with the likelihood of SAMM below 30% as uncomplicated, those with likelihoods between 30-90% as morbid non-SAMM cases and those with likelihoods above 90% as SAMM, the overall proportion of SAMM was similar between InterSAMM and clinicians (the total proportion of deliveries above 90% likelihood of SAMM was 32%), although, based on clinical criteria used, none of the sample were classified as morbid non-SAMM cases by clinicians.

**Figure 3:**
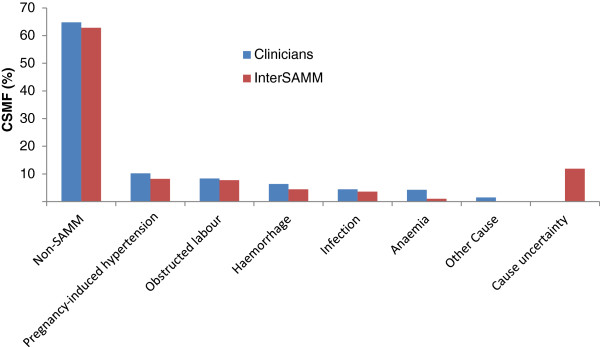
**Distribution of aggregated**^**a**^** broad cause categories for severe acute maternal morbidity (SAMM) cases according to clinician diagnoses and the InterSAMM probabilistic method for 1734 deliveries in Benin and Burkina Faso.** Indeterminate cause CSMFs of 0.1% (InterSAMM) and 0% (clinicians) omitted from the graph.

Given that clinicians only assigned causes to those identified as SAMM cases, comparison of cause distributions are presented with and without the InterSAMM-derived causes of non-SAMM morbid cases (Table 3). With few exceptions, overall cause-specific morbidity fractions were similar in every cause category and population distributions of causes compared well. When aggregated into broad cause-categories of similar aetiologies^a^, the rank order of causes for SAMM cases (>90% likelihood) was identical (Figure 3).

**Table 3:** Population severe acute maternal morbidity (SAMM) cause distributions according to clinician classifications and probabilistic InterSAMM interpretation of data from 1734 deliveries in Benin and Burkina Faso

**Cause**	**Clinician classification**	**InterSAMM method**	
		SAMM* > 90%	SAMM* > 30%
Non-near-miss morbid cases^+^	NA*	19.7%	NA
Uncomplicated	64.8%	43.1%	41.4%
Cause uncertainty^+^	NA	11.9%	19.8%
Uterine rupture/pre-rupture	6.6%	6.2%	6.1%
Post-partum haemorrhage	6.0%	4.3%	4.3%
Pre-Eclampsia	5.4%	3.3%	5.5%
Eclampsia	4.8%	4.9%	5.8%
Anaemia	4.3%	1.1%	4.1%
Genital infection	3.0%	3.3%	8.0%
Obstructed labour	1.8%	1.6%	2.5%
Other cause^++^	1.5%	NA	NA
Malaria	0.8%	0.4%	1.3%
Other infection	0.6%	0.0%	NA
Ante-partum Haemorrhage	0.4%	0.2%	0.2%
Indeterminate cause	0.0%	0.1%	1.1%
Mean (min, max) absolute difference in determinate causes compared to clinician diagnoses		0.9%	1.1%
		(0.1%, 3.2%)	(0.1%, 5.0%)

*SAMM = Severe Acute Maternal Morbidity; NA = Not applicable.

+Cause category for InterSAMM only; ++Cause category for clinicians only.

Of the 1123 cases identified as uncomplicated deliveries by physicians, 25% were identified as morbid non-SAMM cases and 6% as SAMM cases by InterSAMM (Table 3). The majority of causes in these discrepant cases were puerperal infections (24%), with the remainder distributed between pre-eclampsia (14%), anaemia (12%), indeterminate causes (5%), malaria (4%) and obstructed labour (4%). The proportion of uncertainty was 38% for these cases.

### Evaluation

Data were available for 49 of the InterSAMM indicators in the evaluation Benin dataset. The average number of indicators per case was 10 (minimum 3; maximum 22). Data from Bangladesh and India provided information for 34 and 32 indicators, respectively. The average number of indicators was 8 (minimum 4; maximum 16) in the Bangladesh data and 8 (minimum 5; maximum 13) in the India data.

Of the 381 deliveries from Benin, 192 (50%) were classified by physicians as SAMM cases, 63 (17%) were classified as morbid non-SAMM cases and 126 (33%) were classified as uncomplicated. InterSAMM-derived population likelihoods of SAMM were heavily skewed towards the severe end of the scale (not shown) and, using the same cut-offs as above, Table 4 shows the InterSAMM-and physician-derived distribution of morbidity status. Physician classifications of cases from Bangladesh and India did not distinguish between different severities of morbidity and simply classified cases as either morbid or non-morbid. As such, physician review classified 88% and 84% of the cases as morbid in Bangladesh and India, respectively, compared to 56% and 79% according to InterSAMM.

**Table 4:** Distribution and agreement between the InterVA- and clinician-derived morbidity status categories for 381 deliveries from Benin, 57 deliveries from Bangladesh and 51 deliveries from India

**Data source**	**InterSAMM morbidity status categorisation**	**Clinician classification**		
		**Uncomplicated**	**Morbid non-SAMM**	SAMM*
Benin	Uncomplicated	27%	0	0
	Morbid	48%	10%	11%
	non-SAMM			
	SAMM	25%	90%	89%
		**Uncomplicated**	**Morbid**	
Bangladesh	Uncomplicated	28%	12%	
	Morbid	30%	88%	
	non-SAMM			
	SAMM	26%		
India	Uncomplicated	22%	16%	
	Morbid	18%	84%	
	non-SAMM			
	SAMM	61%		
Total	Uncomplicated	25%	14%	
	Morbid	22%	86%	
	non-SAMM			
	SAMM	53%		

*SAMM = Severe Acute Maternal Morbidity.

The major source of discrepancy with regard to SAMM status in the Benin data was among cases classified by physicians as uncomplicated, 48% of which were determined by the probabilistic method to have a non-SAMM morbidity and 25% determined to have been SAMM cases. Causes in these discrepant cases were obstructed labour (17%), anaemia (11%), pre-eclampsia (7%), puerperal infections (7%), post-partum haemorrhage (4%) and indeterminate causes (1%). The proportion attributed to uncertainty was 53%. Scrutiny of the indicators reported in these cases revealed that 57% of cases reported at least one morbidity-specific indicator and almost 13% reported 3 or more (Table 5).

**Table 5:** Frequency of reported indicators among 126 deliveries in Benin classified as uncomplicated by clinicians

**INDICATORS**	**Frequency of positive answers (% among clinician-assigned 'uncomplicated’ cases)**
Antibiotics received	1 (0.8%)
Acute fever ante-partum	4 (3.2%)
Acute fever post-partum	5 (4.0%)
Fever with shivering	9 (7.1%)
Diagnosis of anaemia	18 (14.3%)
Baby’s position abnormal	3 (2.4%)
Major bleeding in early pregnancy	7 (5.6%)
Major bleeding in late pregnancy	1 (0.8%)
Major bleeding during labour	4 (3.2%)
Major bleeding after delivery	14 (11.1%)
Blood pressure raised during pregnancy	5 (4.0%)
Prolonged labour >24 hours	9 (7.1%)
Prolonged labour >48 hours	2 (1.6%)
Delayed delivery of placenta	2 (1.6%)
Manual removal of the placenta	2 (1.6%)
Proteinurea	14 (11.1%)
Referral from one health centre to another	2 (1.6%)
Smelly vaginal discharge	6 (4.8%)
Diagnosis of hypertension	5 (4.0%)
Diagnosis of infection	1 (0.8%)
Self-reported delivery complication	21 (16.7%)
NUMBER OF MORBIDITY INDICATORS	
0	54 (42.9%)
1	37 (29.3%)
2	19 (15.1%)
3+	16 (12.7%)

Though not as consistently close as in the training data, overall cause-specific morbidity fractions were comparable in most cause categories and population distributions of causes generally compared well and are plausible for each setting, both independently and when data were pooled (Table 6).

**Table 6:** Population obstetric morbidity cause distributions of diagnoses by clinicians and probabilistic interpretation of data from 381 deliveries in Benin, 57 in Bangladesh and 51 in India

	**Benin**		**Bangladesh**		**India**		**Total**	
**Cause**	**Clinician diagnosis**	**InterSAMM**	**Clinician diagnosis**	**InterSAMM**	**Clinician diagnosis**	**InterSAMM**	**Clinician diagnosis**	**InterSAMM**
Obstructed labour	34.2%	29.3%	23.5%	10.6%	8.5%	9.4%	30.3%	25.0%
Haemorrhage	10.9%	8.0%	14.2%	8.3%	12.4%	5.6%	11.4%	7.8%
Pregnancy-induced hypertension	7.5%	10.7%	12.5%	19.3%	10.8%	11%	8.4%	11.7%
Infection	13.5%	2.6%	22.7%	9.0%	22.1%	17.1%	15.4%	4.8%
Malaria	1.1%	0%	2.5%	1.5%	10.5%	5%	2.2%	0.7%
Anaemia	4.9%	5.4%	7.1%	5.4%	18.1%	4.9%	6.5%	5.3%
Other cause	1.1%	NA	0%	NA	0%	NA	0.8%	NA
Indeterminate	0%	0.6%	5.3%	0%	2.0%	0%	0.8%	0.5%
Uncomplicated	26.9%	7.1%	12.3%	28%	15.7%	21.6%	24%	11.0%
Cause uncertainty	NA	34.0%	NA	17.9%	NA	25.5%	NA	33.1%
Mean (min, max) absolute difference in determinate causes compared to clinician diagnoses		3.4% (0.5%, 10.9%)		6.8% (1.0%, 13.7%)		4.8% (0.2%, 13.2%)		3.7% (0.3%, 10.6%)

## Discussion

The preliminary development and evaluation of the InterSAMM probabilistic method to estimate the burden and causes of SAMM from community-based surveys of women’s health and pregnancy experiences has highlighted potential strengths and important weaknesses of the approach. Through development and refinement of the method using training data from two settings, it was possible to produce a model that yielded population-level SAMM distributions similar to clinical assessment of the same data. When applied to different test datasets, the probabilistic method compared considerably less well with clinical classification.

Comparisons between probability-derived classifications with quantified uncertainty, and clinician classifications, which only provide absolute positive or negative diagnoses and no measure of uncertainty, is not straightforward. A probabilistic diagnosis of, say, postpartum haemorrhage with 60% likelihood (or 40% uncertainty) is not directly comparable to a physician diagnosis of the same cause with no measureable sense of certainty. Whilst clinical classifications are focused on individual cases and any uncertainty is lost when a final diagnosis is given, the quantified uncertainty of any probabilistic diagnoses can be carried over into the analysis, where the ultimate goal is to estimate population-level burdens of ill health. These differences between the two approaches to interpretation must be kept in mind. Rather than seeking to replicate the exact distributions of SAMM cases and causes derived by clinicians, evaluation focussed on achieving plausible distributions of SAMM and non-SAMM cases and cause distributions that adequately represent burdens of morbidity at the population-level and would be equally as valuable in guiding policy or intervention decisions.

Discrepancies between InterSAMM and clinical classifications highlight potential weaknesses of the current model, for which further thought and revision is needed. For example, when using the test data from Benin, InterSAMM identified considerably more morbid cases than clinicians. This is the crucial common problem with the analysis of self-reported maternal morbidity data and obviously the preliminary InterSAMM method has not overcome problems of low specificity. However, scrutiny of discrepant cases, in which the probabilistic method identified morbidity but clinicians did not, shows that the majority reported symptoms suggestive of some degree of complication. As such, the conclusions reached by the probabilistic method may not be unreasonable from a purely symptom-based probability approach. The challenge remains, however, to move beyond this purely symptom-based approach to improve specificity by further understanding the uncertainty of self-reported symptoms and building this uncertainty, as well as measures of severity, into InterSAMM.

The fact that physicians categorised certain cases as uncomplicated, despite morbidity indicators being reported (Table 5), reflects a divergence in women’s perceptions of childbirth from medical diagnoses of normal labour. This is not surprising, perhaps, but is important from the point of view of population-level measurement from community-based surveys. Clinicians in the Benin and Burkina Faso datasets used strict diagnostic protocols to interpret medical records written by other clinicians to reach a diagnosis. There may have been information available in the hospital records that did not form part of the clinician’s diagnostic criteria for complications, yet may have been used by the probabilistic method. Clinician review of the data from Bangladesh and India did not employ strict diagnostic criteria, with the coding physician being free to diagnose as many potential causes of morbidity as they deemed appropriate from the available interview data, which, in contrast to the West African setting, resulted in more cases being classified as morbid by the clinician than by the probabilistic method. The Benin and Burkina Faso clinicians are also likely to have utilised diagnostic criteria such as blood pressure measurements and clinical observations that are not part of the probabilistic method’s input indicators. Furthermore, symptoms may have been absent from hospital records, particularly if they occurred antepartum or post-discharge, and so were not available for clinical diagnoses but were available to the probabilistic method. These factors may further explain observed discrepancies and may highlight a need to further align InterSAMM with clinical criteria.

Cause-specific discrepancies, such as the varying proportions of infection in the test datasets, may relate to varying definitions of such complications and also to the fact that broad physical symptoms that may be experienced by women during childbirth or postpartum, including shivering or vaginal discharge, can affect the specificity of diagnosis. Greater understanding of the way that clinicians interpreted and valued certain indicators, and how or why clinicians and/or the diagnostic criteria they used excluded certain reported signs and symptoms as indications of morbidity, may be helpful in future refinements of InterSAMM. Similarly, insight into reporting biases in community-based maternal morbidity surveys may inform future refinements. Such information is likely to be useful in establishing *a priori* probabilities for indicators which are frequently over-or mis-reported and should therefore have a lesser influence on raising the likelihood of complications and their causes.

The fact that absolute differences between CSMFs for specific causes varied between settings highlights the problem of variability of diagnoses between settings and coding clinicians as, given the completely standardised way in which the automated probabilistic method handles symptom data, one would expect absolute differences to be fairly consistent if all other factors were held constant. A degree of inter-and intra-rater variability in diagnosing morbidities is inevitable–indeed it is part of the motivation for the development of a standardised method–but it means that comparisons of new methods against inconsistent and potentially flawed reference standards (in which the absolute “gold-standard” diagnoses are difficult to obtain) must be interpreted with caution. Variability in the tools used to collect symptom data from women may have further limited the comparability of results from different settings, as may varying degrees of recall and reporting bias whereby respondents’ answers may be influenced by recollection of events and the perceived desirability of answers [11]. Whilst the probabilistic approach used by InterSAMM may be better able to handle uncertainty in reported indicators due to recall or reporting bias than non-probabilistic methods, any future developments of InterSAMM may need to consider the effect of differing data capture processes and questionnaires and the effect of differing availability of indicators.

Previous work on verbal autopsies has shown that the probabilistic InterVA method for cause of death ascertainment is relatively insensitive to minor variations in the prior probabilities [16]. The same is likely to be true for the probabilistic method applied to maternal morbidity in this study, and may explain why “ball-park” probabilities in the current model and refinements using the hospital-based, training datasets from Benin and Burkina Faso, were sufficient to create a workable model to explore the method’s potential utility. Nevertheless, should the method be developed further, a more sound approach to establishing *a priori* probabilities should be used. Work on verbal autopsies has successfully used a system of expert consensus to approximate underlying probabilities [12,17,18], and, given the lack of existing, reliable data on the burden and causes of obstetric morbidity in communities where many women deliver at home, a similar approach may be appropriate for InterSAMM. Further understanding of how women perceive and describe morbidity symptoms and delivery complications could also benefit the development of the probability matrix and the indicators used, perhaps involving input from women themselves or birth attendants. Finally, there may be a need for contextual variations in *a priori* probabilities where, rather than a protective factor, for example, delivery at a facility may indicate a complication in a population that normally delivers at home.

None of the data used during the development and preliminary evaluation of InterSAMM are representative of a general population of recently-delivered women, in which one would expect the vast majority to have had uncomplicated pregnancies, deliveries and post-partum periods. InterSAMM’s performance in a population for which it is intended remains untested. Further testing on data from a range of settings is important, although sourcing suitable datasets with adequate reference diagnoses for comparison has proved challenging. More detailed explorations of diagnostic accuracy at the individual and population level, such as sensitivity, specificity and positive predictive values, or assessments of inter-rater agreement, may be appropriate in future evaluations, but, once again, their interpretation must be grounded in the realities of the reference standards being used. Newly proposed chance-corrected concordance and diagnostic accuracy measures that take into consideration the potential for random agreement between methods that can generate multiple causes from a finite list of causes may also be useful in future evaluations [19]. All evaluations, however, must relate to the intended use of InterSAMM to estimate population levels of morbidity and its causes, whereby shortcomings in accuracy may be offset by plausibility, efficiency, adequacy for purpose and advantages of unique reliability. There may also be a need for a compromise between strict diagnostic criteria, as would be used in clinical settings, and broader conceptualisation of morbidity as deemed important by women themselves and important to population-level understandings of SAMM to inform public health.

## Conclusion

The preliminary probabilistic InterSAMM method described and evaluated here has important limitations, but shows promise in overcoming longstanding barriers to standardised measurement of maternal morbidity that allows for the uncertainty of women’s self-reported symptoms in retrospective interviews. Further development, refinement and evaluation, as well as exploration of other statistical methods [20-22], is likely to be worthwhile for its potential to advance the measurement of maternal morbidity and revealing population burdens and causes of severe acute maternal morbidity.

## Endnote

^a^Aggregated broad cause categories were as follows: Pre-eclampsia/Eclampsia = Pregnancy Induced Hypertension (PIH); Uterine rupture/pre-rupture/Obstructed labour = Dystocia; Ante-partum/post-partum haemorrhage = Haemorrhage; Genital infection/Malaria/Other infection = Infection.

## Abbreviations

SAMM: Severe Acute Maternal Morbidity; InterSAMM: Interpreting Severe Acute Maternal Morbidity; CSMF: Cause Specific Morbidity Fraction.

## Competing interests

The authors declare they have no competing interests.

## Authors’ contributions

EF designed the study, developed InterSAMM, conducted analyses and wrote the first draft of the manuscript. UH and CR contributed to the development and refinement of InterSAMM, interpretation of results, drafting and revising the manuscript. DO contributed to the analysis and interpretation of data from India and Bangladesh and revisions of the manuscript. KA, NN, NM, RG and SG contributed original data, technical input, interpretation and revisions of the manuscript. PB developed the original computer program on which InterSAMM is based, provided technical oversight to the development and evaluation of InterSAMM and contributed to the interpretation of results and revisions to the manuscript. VF provided technical oversight of the whole project and was directly involved in the generation, analysis and interpretation of data from West Africa, and drafting and revising the final manuscript. VP also secured the CHERG funding to support this work. All authors have read and approve the final manuscript.
